# Evaporation‐Driven Solutal Marangoni Control of Rayleigh–Taylor Instability in Inverted Films

**DOI:** 10.1002/advs.202520343

**Published:** 2026-01-29

**Authors:** Minwoo Choi, Hyejoon Jun, Hyoungsoo Kim

**Affiliations:** ^1^ Department of Mechanical Engineering KAIST Daejeon South Korea

**Keywords:** Rayleigh‐Taylor instability, selective evaporation, solutal Marangoni effect, thin‐film stability control

## Abstract

Inverted liquid films are inherently unstable: gravity amplifies perturbations through the Rayleigh–Taylor instability (RTI), causing rupture and dripping. We show that selective evaporation in volatile binary mixtures can suppress, delay, or transform this instability by inducing solutal Marangoni stresses. Systematic experiments and a complementary theoretical model reveal three instability regimes— promotion, suppression, and sustained oscillations—set by volatility, viscosity, and surfacetension contrast. High‐resolution deflectometry tracks spatiotemporal film evolution, while linear stability analysis captures the competition among gravity, capillarity, and Marangoni forces. Measurements and theoretical predictions agree well quantitatively, confirming the mechanism and establishing evaporation‐driven Marangoni flow as a robust interfacial control strategy for inverted films. Our results deliver the first systematic experimental validation of solutal‐Marangoni suppression of RTI in inverted configurations and provide a general framework for instability control in volatile thin films, with implications for materials processing, coating, and soft‐matter hydrodynamics.

## Graphical abstract

Inverted liquid films, like paint on a ceiling, are inherently unstable under gravity. This work shows that selective evaporation in volatile binary mixtures generates solutal Marangoni stresses that either suppress or amplify the Rayleigh Taylor instability. Experiments and theory identify three regimes enhanced growth, suppression, and sustained oscillations, establishing evaporation driven Marangoni flow as a passive strategy for controlling dripping in inverted thin films.

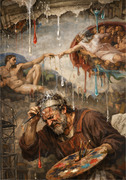

## 1 Introduction

**FIGURE 1:**
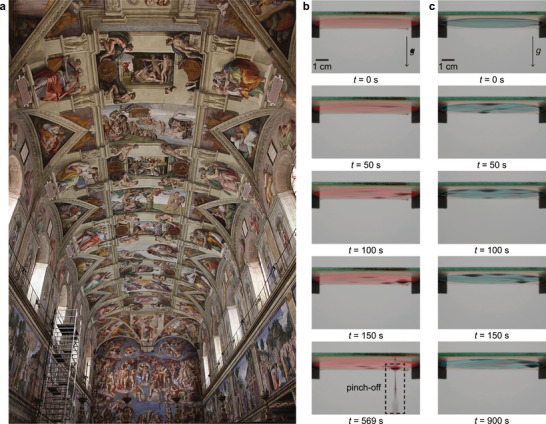
Illustration and experimental demonstration of inverted thin films. a) *The Creation of Adam* by Michelangelo (1511), painted on the ceiling of the Sistine Chapel. This iconic artwork exemplifies the practical challenges of stabilizing inverted films against gravity. Photograph by Jörg Bittner Unna, licensed under CC BY 3.0 (https://creativecommons.org/licenses/by/3.0/). Source: Wikimedia Commons (https://commons.wikimedia.org/wiki/File:%27;Sistine_Chapel_ceiling%27;_by_Michelangelo_JBU21.JPG). b) Time‐lapse snapshots showing the destabilization of a red‐pigmented aqueous film (80 wt% water mixed with 20 wt% red pigment) on an inverted glass substrate (initial film thickness, $h_{0}=150\;\mu m$). A gravity‐driven pinch‐off event is observed at t=569s. c) A mixture of 72 wt% water and 8 wt% ethanol with 20 wt% green pigment ($h_{0}=150\;\mu m$) remains stable up to 900 s, indicating suppression of the Rayleigh–Taylor instability. For additional experimental details and video visualization, see Video S1.

Imagine Michelangelo, lying on his back beneath the Sistine Chapel ceiling, painstakingly painting the masterpiece of *The Creation of Adam*, as shown in Figure 1a. Each brushstroke formed a thin liquid film on the overhead surface—an inherently unstable configuration held against gravity. The ceiling thus represents an early, real‐world example of an inverted film where dripping had to be avoided. This physical challenge, likely a source of delay in completing the fresco [1], is governed by the Rayleigh–Taylor instability (RTI), which arises when a denser fluid is suspended above a lighter one under gravity. In this case, the paint layer was prone to RTI‐induced rupture.

The RTI is a fundamental hydrodynamic instability that has been extensively studied, both experimentally and theoretically since the nineteenth century, due to its significance in numerous natural and technological contexts [2, 3, 4, 5, 6]. A well‐known phenomenon is the destabilization of inverted thin liquid films beneath flat surfaces, which eventually rupture and drip due to gravitational effects. To suppress RTI in such films, several stabilization strategies have been proposed—including vertical oscillations [7], electric fields [8], thermal gradients [9, 10], and geometric confinement [11]—mostly focusing on pure, nonvolatile liquids.

In contrast, many real‐world applications, such as painting and coating, involve volatile or multicomponent liquids. These introduce additional complexities including evaporation, concentration gradients, and interfacial stresses, which are classified as chemical Marangoni effects [12]. Prior investigations of evaporation‐induced Marangoni effects have largely been conducted for on‐plate films on substrates [13, 14], whose base states are gravitationally stable. Accordingly, suppression has typically been discussed in terms of capillary‐scale undulations rather than the Rayleigh–Taylor rupture that threatens inverted layers. Related theoretical studies on upright films have further predicted oscillatory responses arising from competing thermal and solutal Marangoni effects [15, 16, 17].

Despite these advances, an inverted thin film beneath a rigid substrate—an intrinsically unstable configuration– ‐ has not been systematically resolved regarding regime transitions and complete suppression of dripping, nor has there been systematic experimental verification under controlled liquid composition. While RT behavior in evaporating binary mixtures has been analyzed [18], solutal Marangoni effects have typically been regarded as a separate and potentially uncontrollable source of Marangoni instability rather than as a mechanism for mitigating instability. This raises a fundamental and practical question: Can solutal Marangoni effect suppress or modify the classical RTI in inverted films under control?

To address this, we experimentally examined the dynamics of binary liquid films hanging beneath a flat plate, focusing on how evaporation‐driven Marangoni flows influence their stability. As a proof of concept (Figure 1b,c), we mimicked ceiling painting using water‐based solutions. A film composed of pure water (80 wt%) and red pigment (20 wt%) ruptured and dripped at t=570s (Figure 1b). In contrast, adding a small amount of ethanol (8 wt%) to the water (72 wt%) completely suppressed dripping (Figure 1c), allowing the film to remain intact until full evaporation. This demonstrates that introducing a volatile component like ethanol triggers solutal Marangoni stresses that passively suppress the instability—suggesting a simple and energy‐free mechanism that could help mitigate practical challenges, including those faced by Michelangelo during his legendary ceiling painting.

To understand this result, we combined experimental and theoretical approaches to investigate the instability of binary mixture thin films under a substrate. Using deflectometry to monitor spatial variations in film thickness (see Note S2, Supporting Information), we found that the system exhibited three distinct instability behaviors—enhancement of the growth rate, suppression, and oscillation—depending on the mixture composition. These regimes are governed by solutal Marangoni stresses arising from selective evaporation. Furthermore, linear stability analysis yielded analytical models that describe the observed modes, including growing, suppressing, and oscillatory behaviors. These models, supported by experimental data, demonstrate the pivotal role of solutal Marangoni flows in regulating RTI across different regimes.

## 2 Results and Discussion

### 2.1 Mechanisms of Solutal Marangoni‐Driven Suppression and Promotion

**FIGURE 2:**
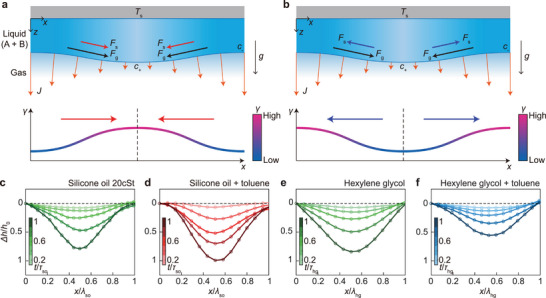
Control of the Rayleigh–Taylor instability in binary mixtures based on surface tension gradients between two components. (a, and b) Schematic of an interfacial tension (γ) profile in a binary mixture. In (a), the more volatile liquid B has a higher surface tension (Δγ<0), whereas in (b), it has a lower surface tension (Δγ>0). For (a) and (b), $F_{s}$ and $F_{g}$ are the solutal Marangoni and gravity‐driven forces, respectively, and the orange arrows represent the evaporative mass flux. (c–f) Evolution of thin film profiles of silicone oil 20 cSt (c), hexylene glycol (e), silicone oil and toluene (Δγ<0) (d), and hexylene glycol and toluene (Δγ>0) (f). All variables are dimensionless, namely, $z=z/h_{0}$, x=x/λ, and t=t/τ, where $h_{0}=80\;\mu m$ is the initial film thickness, λ is the most unstable wavelength and τ is the most unstable time scale of the nonvolatile component in the mixture. The subscripts “so” and “hg” denote pure silicone oil 20 cSt and hexylene glycol, respectively.

We observed that in a binary mixture system, the selective evaporation plays a dominant role in governing whether the instability is suppressed or promoted in the inverted thin film. The mixtures consisted of a nonvolatile component A and a volatile component B, and were studied under room temperature conditions in the inverted configuration, as illustrated in Figure 2a,b. During evaporation, the local evaporation rate (J indicated by orange arrows in Figure 2a,b) is typically higher at thinner regions (crests) than at thicker regions (troughs)[19, 20]. This phenomenon is attributed to the vertical heat transfer through the liquid film, as evaporation is an endothermic process requiring latent heat supplied from the substrate. Thinner regions exhibit a lower thermal resistance, leading to a higher local interfacial temperature, which in turn enhances the local evaporation rate.

This differential evaporation enriches the volatile component B in the troughs, leading to a spatial gradient in the mass fraction of B, denoted by c at the trough. In the presence of a surface tension contrast between the two pure components, this compositional nonuniformity gives rise to a local surface tension gradient along the interface. To determine whether the resulting solutal Marangoni effect promotes or suppresses the instability, the direction of this surface tension gradient is crucial. We therefore define $\Delta \gamma \equiv \gamma_{A}-\gamma_{B}$, where $\gamma_{A}$ and $\gamma_{B}$ are the surface tensions of the nonvolatile (A) and volatile (B) components, respectively. If $\gamma_{B}>\gamma_{A}$ (i.e., Δγ<0), the induced solutal Marangoni stresses act from crests toward the troughs, enhancing the instability if $\gamma_{B}<\gamma_{A}$ (Δγ>0), the Marangoni stresses oppose the gravitational destabilization, thereby suppressing the instability.

### 2.2 Experimental Measurement of Instability Evolution

To verify these two cases, we conducted experiments to measure the evolution of the film interface profiles of various binary mixture thin films over time, using deflectometry (details in Supporting Information, Section 2). The film thickness differences Δh(x,t) [$=h\left(x,t\right)-h_{0}$] were measured, where h(x,t) was the local film thickness, and $h_{0}=80\;\mu m$ was the initial film thickness. We compared the temporal thickness evolution of a nonvolatile pure liquid film and a binary liquid film, in which a volatile component was mixed with the same liquid, under the substrate (Figure 2c–f; Videos S2 and S3, Supporting Information). The film thickness, time, and horizontal length were nondimensionalized by the initial thickness, the most unstable time scale, and wavelength of the pure liquid, respectively. It was found that when Δγ<0 (90 wt% silicone oil 20 cSt and 10 wt% toluene), the instability in the suspended thin film became promoted (Figure 2c,d), while when Δγ>0 (90 wt% hexylene glycol and 10 wt% toluene), it was suppressed (Figure 2e,f).

### 2.3 Experimental Analysis of the Oscillatory Mode

**FIGURE 3:**
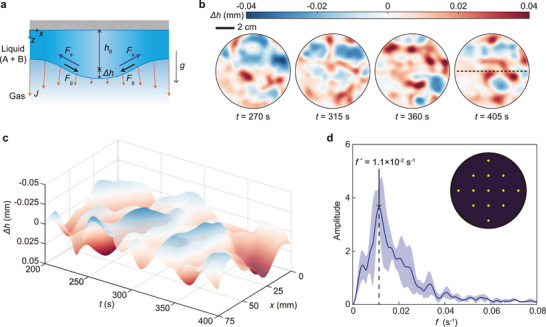
Oscillatory mode of Rayleigh‐Taylor instability in a mixture of 80 wt% ethylene glycol and 20 wt% 2‐butanol. a) Schematic illustration of the instability in an evaporating thin liquid film composed of A and B, where $h_{0}$ and Δh denote the initial film thickness and the variation in film thickness along the z‐axis, respectively. The orange arrows represent the evaporative mass flux. b) Time evolution of the thin film thickness in a two‐dimensional area was measured using deflectometry, where the diameter of the substrate, 2r = 8cm. c) Time evolution of the thin film thickness along the black dashed line (length = 7.5cm, almost one diameter) as indicated in (b). d) Fast Fourier transform (FFT) of the film thickness at representative 13 points (see the yellow dots in the inset). The blue solid line indicated the averaged FFT results of 13 points and the blue‐shaded area represented the error ranges at each frequency, determined from the standard deviation of the 13 points.

An oscillatory mode was observed when the solutal Marangoni‐driven force ($F_{s}$) opposed and exceeded the gravity‐driven force ($F_{g}$), as illustrated in Figure 3a. This competition between stabilizing and destabilizing forces led to periodic deformation of the film interface. Figure 3b,c shows experimental results for the oscillatory mode in a binary mixture thin film (80 wt% ethylene glycol and 20 wt% 2‐butanol) suspended beneath a flat plate at room temperature. A two‐dimensional contour map in Figure 3b presents the time evolution of the film thickness from t=200s, when initial perturbations had developed across the entire film, to t=500s, by which time the volatile component had nearly evaporated. During this interval, exponential growth in surface deformation was observed (see Video S4, Supporting Information). Periodic fluctuations in film thickness (Δh) were captured along the dashed horizontal line in Figure 3b and are plotted in Figure 3c, clearly showing oscillatory behavior. A fast Fourier transform (FFT) analysis of the spatiotemporal film thickness (Figure 3d) confirmed this periodicity, revealing a dominant frequency of ($f=1.1\times 10^{-2}\;s^{-1}$). The time‐series data used for FFT were extracted from the yellow points indicated in the inset of Figure 3d (see also Note S3, Supporting Information).

### 2.4 Analytical Validation of the Oscillatory Mode

To validate our experimental findings and gain deeper insight into the underlying mechanisms, we developed a theoretical model based on linear stability analysis. The model captures the competition between gravity and solutal Marangoni forces that influence the Rayleigh–Taylor instability (RTI) in evaporating binary films. As the suspended binary film evaporates, the mass fraction of the volatile component varies spatially due to local differences in the evaporation rate, which depends on the local film thickness, h(x,t). This nonuniform evaporation generates in‐plane surface tension gradients, leading to solutal Marangoni stresses. For the vertical distribution of concentration, we employed the vertical averaging (VA) approximation [21, 22], in which the local mass fraction c(x,z,t) is decomposed into a depth‐averaged component C(x,t) and a small perturbation $C_{1}\left(x,z,t\right)$. This approximation allows us to assume ∂c/∂z≈0 (see Supporting Information, Section 1). The local surface tension (γ) was defined as a linear function of the mass fraction of the volatile component: $\gamma =\gamma_{A}\left(1-c\right)+\gamma_{B}c$ [18, 23]. Consequently, the tangential stress balance at the interface gives $\rho \nu u_{z}=\partial \gamma /\partial x=-\Delta \gamma c_{x}$, where ρ is the density and ν is the kinematic viscosity. In this case, the thermal Marangoni stress was relatively less important in the presence of a phase change for a single component [24] as well as binary mixtures [25]. The diffusion coefficient (D) between two components was estimated using the Stokes‐Einstein relation [26]. Assuming the film was sufficiently thin, i.e., $\varepsilon (=h_{0}/\lambda )\ll 1$ (with $h_{0}$ the initial film thickness and λ the horizontal length scale), the lubrication approximation was applied [20]. Under these conditions, the buoyancy‐driven convection was neglected [27] due to a small Rayleigh number, estimated as $\text{Ra}=g\beta \Delta Th_{0}^{3}/\nu \kappa =O\left(10^{-9}\right)-O\left(10^{-5}\right)$, where g is the gravitational acceleration, β is the thermal expansion coefficient, $\Delta T=T_{s}-T_{g}$ is the temperature difference between the substrate ($T_{s}$) and the gas phase ($T_{g}$), and κ is the thermal diffusivity.

During evaporation, we assumed that the binary mixture has constant thermophysical properties, determined from the mass averaged mixing rule using the initial mass fraction of component B ($c_{0}$) because the Biot number is much smaller than unity, $\text{Bi}=C_{h}h_{0}/k=O\left(10^{-3}\right)-O\left(10^{-2}\right)$, where $C_{h}\approx 10-100\;W/m^{2}K$ is the typical heat transfer coefficient of the air [28], and k is the thermal conductivity of the film. Although the film can be considered isothermal under these Biot number conditions when the film thickness is locally uniform, spatial variations in film thickness along the horizontal direction lead to local differences in evaporation rate due to the large aspect ratio. Under these conditions, the Hertz‐Knudsen equation could be applied as $J=cp_{0}L{\left(M^{3}/2\pi R_{g}^{3}T_{g}^{5}\right)}^{1/2}\left(T{|}_{h}-T_{g}\right)$ at the liquid‐gas interface [19], where $p_{0}$ is the vapor pressure of the component B, L is the specific latent heat of vaporization of the component B, M is the molecular weight of the component B, and $R_{g}$ is the universal gas constant.

Based on the above assumptions, we obtained the dimensionless evolution equations for the film thickness and the mass fraction by rescaling $\lambda \to h_{0}$ (ε=1) (the details of the derivation in Note S1, Supporting Information):

(1) $${\tilde{h}}_{\tilde{t}}=-\frac{EC}{K+\tilde{h}C}+{\left[\frac{{\tilde{h}}^{3}}{3}\left(-\frac{{\tilde{h}}_{\tilde{x}\tilde{x}\tilde{x}}}{\text{Ca}}-\text{Ga}{\tilde{h}}_{\tilde{x}}\right)+\frac{\text{Ma}}{2\text{Pr}}{\tilde{h}}^{2}C_{\tilde{x}}\right]}_{\tilde{x}},$$

(2) $$C_{\tilde{t}}=\frac{EC(C-1)}{K+\tilde{h}C}+\frac{{\left(\tilde{h}C_{\tilde{x}}\right)}_{\tilde{x}}}{\tilde{h}\text{Pe}}+\left[\frac{{\tilde{h}}^{2}}{3}\left(-\frac{{\tilde{h}}_{\tilde{x}\tilde{x}\tilde{x}}}{\text{Ca}}-\text{Ga}{\tilde{h}}_{\tilde{x}}\right)+\frac{\text{Ma}}{2\text{Pr}}\tilde{h}C_{\tilde{x}}\right]C_{\tilde{x}},$$

where E=kΔT/ρνL is the evaporation number, $K=\left(k/h_{0}L^{2}p_{0}\right){\left(2\pi R_{g}^{3}T_{g}^{5}/M^{3}\right)}^{1/2}$ is the degree of nonequilibrium at the evaporating interface, $\text{Ca}=\rho \nu^{2}/\gamma_{0}h_{0}$ is the capillary number, $\text{Ga}=gh_{0}^{3}/\nu^{2}$ is the Galileo number, $\text{Ma}=\Delta \gamma h_{0}/\rho \nu \kappa$ is the solutal Marangoni number, Pr=ν/κ is the Prandtl number, and Pe=ν/D is the Péclet number. From these equations, all nondimensionalized variables will be presented with a tilde (∼). Specifically, the solutal Marangoni number, representing the solutal Marangoni effect at the interface, can be either positive or negative depending on Δγ.

To perform the linear stability analysis, we introduced the film thickness and concentration perturbations of the form $\tilde{h}={\tilde{h}}_{b}+\hat{H}e^{\tilde{\sigma }\tilde{t}+i\tilde{q}\tilde{x}}$ and $C=c_{b}+\hat{C}e^{\tilde{\sigma }\tilde{t}+i\tilde{q}\tilde{x}}$, respectively, where $h_{b}$ and $c_{b}$ are the film thickness and mass fraction at the base state, $\hat{H}$ and $\hat{C}$ are the amplitudes of linear perturbations, and $\tilde{\sigma }$ and $\tilde{q}$ are the corresponding linear growth rate and spatial wavenumber, respectively. For the current problem, we assumed that diffusion dominates over internal convective flow within the thin film, since the evaporative Péclet number is much smaller than unity, ${\text{Pe}}_{e}=U_{e}h_{0}/D=O\left(10^{-6}\right)-O\left(10^{-3}\right)$ [29], where $U_{e}=k\Delta T/\rho h_{0}L$ is the evaporation‐driven flow velocity. Due to this, the base and initial conditions still hold, $h_{b}\approx 1$ and $c_{b}\approx c_{0}$, and therefore an analytical solution for the linear growth rate can be derived simply by only considering the major terms—gravity, capillarity, and solutal Marangoni stresses (details in Note S1, Supporting Information) as:

(3) $$\tilde{\sigma }=\frac{1}{2}\left(\frac{\text{Ga}}{3}{\tilde{q}}^{2}-\frac{1}{3\text{Ca}}{\tilde{q}}^{4}\right)\pm \frac{1}{2}\sqrt{\tilde{\zeta }},$$

where $\tilde{\zeta }={\left(\text{Ga}{\tilde{q}}^{2}/3-{\tilde{q}}^{4}/3\text{Ca}\right)}^{2}-2\left[E\left(1-c_{0}\right)\left(K/c_{0}+2\right)/{\left(K/c_{0}+1\right)}^{2}\right]\left(\text{Ma}/\text{Pr}\right){\tilde{q}}^{2}$ (see more details in Figure S1a,b, Supporting Information). When $\tilde{\zeta }<0$, the linear growth rate becomes complex, indicating oscillatory behavior of the interface over time. The observed frequency (${\tilde{f}}^{*}$) corresponds to the imaginary part of the complex linear growth rate at the most unstable mode, ${\tilde{f}}^{*}=\sqrt{|{\tilde{\zeta }}^{*}|}/2$, which satisfies $d\tilde{\sigma }/d\tilde{q}=0$ at the most unstable wavenumber, ${\tilde{q}}^{*}\approx \sqrt{\text{Ga}\text{Ca}/2}$. The corresponding most unstable linear growth rate decreases as Ma increases and eventually becomes complex beyond a certain value of Ma (${\tilde{\zeta }}^{*}=0$), indicating the transition to the oscillatory mode (Figure S1c, Supporting Information). Physically, the binary film becomes stable when the surface tension of the volatile liquid is lower than that of the nonvolatile liquid. However, increasing the surface tension gradient further causes the solutal Marangoni force to exceed gravitational forces, leading to oscillatory behavior.

**FIGURE 4:**
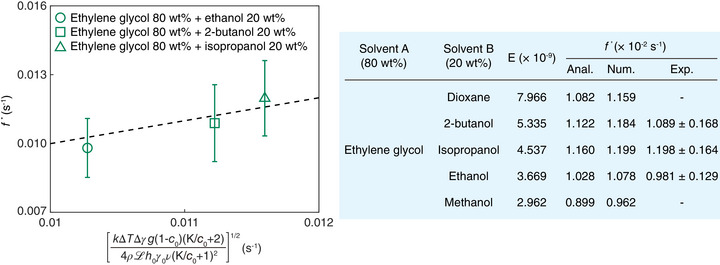
Temporal evolution of thin film thickness in binary mixtures and comparison with theoretical predictions. Frequencies of the oscillating film thickness, compared to the analytical solution. The table shows experimental conditions and the frequencies for the oscillatory instability in binary mixtures at 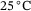 where the initial film thickness is 80μm.

The dimensional frequency ($f^{*}={\tilde{f}}^{*}\nu /h_{0}^{2}$) of this oscillatory mode was experimentally validated using ethylene glycol‐based binary mixtures with various volatile additives, as summarized in Figure 4. Among these, oscillations were observed in three mixtures. Frequencies measured by FFT were compared with the analytical and numerical solutions based on Equations (1) and (2), solved via the finite difference method (Note S5 and Figure S2, Supporting Information). The theoretical predictions show a good agreement with experimental results.

In the experiments, two mixtures that were analytically predicted to exhibit oscillatory modes did not show such behavior. For the ethylene glycol‐dioxane mixture, the volatile component completely evaporated prior to the experimental preparation due to its high evaporation rate (high E), making frequency measurements impossible. Conversely, the ethylene glycol‐methanol mixture, despite having the slowest evaporation rate (low E), exhibited monotonic growth with a reduced linear growth rate compared to pure ethylene glycol.

### 2.5 Linear Dependence of Growth Rate on Marangoni Number under Slow Evaporation

We also found that hexylene glycol‐based mixtures (e.g., hexylene glycol with toluene in Figure 2f), despite having a positive surface tension gradient, exhibited only exponential growth due to an extremely low evaporation number. The instabilities in binary mixture films with evaporation number below $3\times 10^{-9}$ showed no oscillations and were either monotonically suppressed or promoted, where the evaporation number quantifies the ratio of the viscous time scale, $\tau_{v}=h_{0}^{2}/\nu$, to the evaporative time scale, $\tau_{e}=\rho h_{0}^{2}L/k\Delta T$ [19]. Such mixtures typically evaporate slowly and exhibit high viscosity (see Note S4 and Figure S3, Supporting Information). Under these conditions, slow evaporation and dominant viscous dissipation suppress oscillatory flows, rendering transient variations in mass fraction negligible. As a result, the mass fraction for the linear stability analysis in the long‐term limit should be modified to $C=c_{b}+\hat{C}e^{i\tilde{q}\tilde{x}}$, neglecting the linear growth rate of the mass fraction, $\partial C/\partial \tilde{t}=0$. Finally, the linear growth rate for the slowly evaporating mixtures is derived as (details in Note S1, Supporting Information):

(4) $$\tilde{\sigma }=\frac{\text{Ga}}{3}{\tilde{q}}^{2}-\frac{1}{3\text{Ca}}{\tilde{q}}^{4}-\frac{E\text{Pe}\left(1-c_{0}\right)\left(K/c_{0}+2\right)}{2{\left(K/c_{0}+1\right)}^{2}}\frac{\text{Ma}}{\text{Pr}}.$$

Here, the most unstable wavenumber is unchanged, i.e., ${\tilde{q}}^{*}=\sqrt{\text{Ga}\text{Ca}/2}$, while the most unstable linear growth rate (${\tilde{\sigma }}^{*}$) decreases proportionally to the solutal Marangoni number.

**FIGURE 5:**
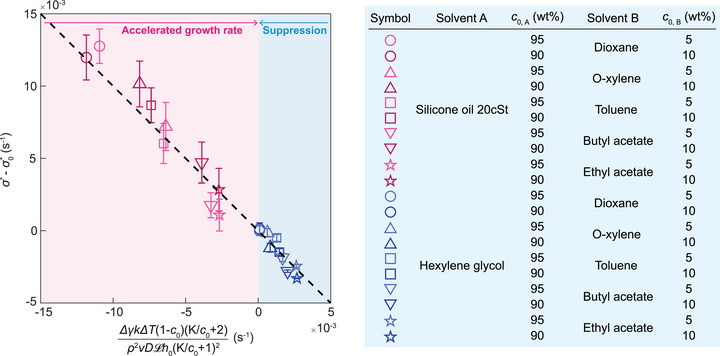
Control of thin film instability through solutal Marangoni effects. The figure presents the variation in linear growth rate ($\sigma^{*}$ − $\sigma_{0}^{*}$) of instability during the exponential growth phase as a function of the surface tension gradient (Δγ). Here, $\sigma^{*}$ and $\sigma_{0}^{*}$ are the linear growth rates with and without the solutal Marangoni effect, respectively. Error bars indicate standard deviations from experimental measurements. The table shows experimental conditions for comparing growth rates of the instability in binary mixtures.

The dimensional linear growth rates ($\sigma^{*}={\tilde{\sigma }}^{*}\nu /h_{0}^{2}$) predicted by Equation 4 were validated by comparison with experimentally measured values. In experiments, the linear growth rate was determined by fitting the time‐dependent film thickness data within the initial growth regime, where exponential behavior dominates before nonlinear effects become significant [30]. To compare the theoretical predictions with the experiments, the linear growth rate and the solutal Marangoni number were rescaled based on Equation (4), where $\sigma_{0}^{*}=\rho^{2}g^{2}h_{0}^{3}/12\mu \gamma$ is the linear growth rate without the solutal Marangoni effect [31]. Extensive experiments conducted on mixtures with the low evaporation number showed excellent agreement with theoretical predictions, as shown in Figure 5, confirming that the instability in the upside down thin film is suppressed as Δγ increases.

## 3 Conclusion

This study reveals that the classical Rayleigh–Taylor instability (RTI), long considered an inevitable consequence in inverted fluid configurations, can be passively suppressed or fundamentally altered through solutal Marangoni effects. By introducing a volatile liquid with lower surface tension into a binary liquid mixture (Δγ>0), we dramatically reduced the linear growth rate to just 6.9% of the pure liquid scenario ($\sigma_{\text{binary}}^{*}/\sigma_{\text{pure}}^{*}=0.069$). Remarkably, when the Marangoni stress exceeded gravitational destabilization in the opposite direction, the interface exhibited sustained oscillatory motion, significantly delaying the onset of exponential instability.

These findings are supported by both experimental observation and linear stability analysis, and culminate in a uniform coating demonstration using a mixture of 36 wt% water, 60 wt% pigment, and 4 wt% ethanol ($\Delta \gamma =50{m\;N\;m}^{-1}$), as shown in Figure S4 (Supporting Information), under conditions where the evaporation number is low, $E=O\left(10^{-11}\right)-O\left(10^{-10}\right)$, and the Marangoni number is large and positive, $\text{Ma}=O\left(10^{2}\right)-O\left(10^{3}\right)$.

Our results challenge conventional RTI mitigation strategies—such as mechanical vibrations, electric fields, and thermal gradients—that are limited to nonvolatile, single‐component systems. In contrast, we introduce a simple, robust, and energy‐free approach based on interfacial design using multicomponent evaporation‐driven flows. Beyond advancing fundamental understanding, this mechanism offers a generalizable design principle for controlling the stability of inverted or accelerated thin films in diverse applications where body forces are significant. These include anti‐sag and anti‐drip architectural coatings [32], conformal coatings on complex vehicle components and assemblies [33], and overhead painting [34], as well as thin film systems subjected to extreme accelerations, such as inertial confinement fusion systems [35].

By enabling predictive and tunable control of interfacial instabilities, this work lays the foundation for next‐generation technologies involving stable yet reconfigurable thin films under inherently unstable configurations.

## 4 Experimental Section

### Sample Preparation

All binary mixtures were prepared by combining a volatile liquid with a nonvolatile liquid, with the mass fraction of the volatile component carefully controlled within the range of 5–20 wt%. This range was chosen to minimize evaporative effects, as predicted by Raoult's law [36], while ensuring that the thermophysical properties of the binary mixtures remained sufficiently stable for experimental consistency.

The nonvolatile liquids used in this study were high‐viscosity fluids to prevent rapid film deformation during experiments. These included ethylene glycol (99.5%, Samchun) (density, $\rho =1.11\;g\;cm^{-3}$, dynamic viscosity, μ=16.1mPas, and surface tension $\gamma =48.0\;mN\;m^{-1}$), hexylene glycol (purity = 99%, Sigma‐Aldrich) ($\rho =0.920\;g\;cm^{-3}$, μ=34.1mPas, and $\gamma =33.1\;mN\;m^{-1}$), and silicone oil 20 cSt (Sigma‐Aldrich) ($\rho =0.949\;g\;cm^{-3}$, μ=19.0mPas, and $\gamma =20.6\;mN\;m^{-1}$). The volatile solvents added to these mixtures were dioxane (anhydrous, 99.8%, Sigma‐Aldrich) ($\rho =1.03\;g\;cm^{-3}$, μ=1.21mPas, and $\gamma =32.8\;mN\;m^{-1}$), 2‐butanol (anhydrous, 99.5%, Sigma‐Aldrich) ($\rho =0.810\;g\;cm^{-3}$, μ=3.25mPas, and $\gamma =24.3\;mN\;m^{-1}$), isopropanol (anhydrous, 99.5%, Sigma‐Aldrich) ($\rho =0.785\;g\;cm^{-3}$, μ=2.04mPas, and $\gamma =20.9\;mN\;m^{-1}$), ethanol (anhydrous, 99.5%, Sigma‐Aldrich) ($\rho =0.789\;g\;cm^{-3}$, μ=1.07mPas, and $\gamma =22.0\;mN\;m^{-1}$), methanol (99.9%, Sigma‐Aldrich) ($\rho =0.792\;g\;cm^{-3}$, μ=0.539mPas, and $\gamma =23.6\;mN\;m^{-1}$), o‐xylene (anhydrous, 99%)($\rho =0.876\;g\;cm^{-3}$, μ=0.747mPas, and $\gamma =29.6\;mN\;m^{-1}$), toluene (anhydrous, 99.8%, Sigma‐Aldrich) ($\rho =0.867\;g\;cm^{-3}$, μ=0.548mPas, and $\gamma =27.7\;mN\;m^{-1}$), butyl acetate (99.5%, Sigma‐Aldrich) ($\rho =0.882\;g\;cm^{-3}$, μ=0.685mPas, and $\gamma =24.8\;mN\;m^{-1}$), and ethyl acetate (99.9%, Sigma‐Aldrich) ($\rho =0.896\;g\;cm^{-3}$, μ=0.423mPas, and $\gamma =23.2\;mN\;m^{-1}$). All properties were measured at 298K. Further details on the thermophysical properties and dimensionless numbers of the prepared mixtures are provided in Note S6 and Tables S1– S3 (Supporting Information).

### Substrate Preparation

Square glass plates (10cm wide and 2±0.01mm thick) were used as substrates. The liquid film was deposited on the circular area with a diameter of 8cm and a thickness of 80μm. This area is bounded by copper tape attached to the glass plates to prevent the liquid from spreading beyond the designated boundary. The diameter (d) of the liquid film should be several times larger than the typical length scale determined by the balance between surface tension and gravity, $d\gg 2\pi \sqrt{2}l_{c}$, where $l_{c}=\sqrt{\gamma /\rho g}$ is the capillary length.

### Film Thickness Measurement

We coated liquid mixtures to the substrate with a volume of V=402μL, ensuring that the initial film thickness ($h_{0}=80\;\mu m$) matched the thickness of the copper tape. To flatten the liquid and minimize the evaporation of solvents, we employed a film applicator and treated the substrate with plasma. After that, this substrate was flipped back over to form a suspended liquid film. In this process, the substrate should be flipped faster than the most unstable time scales of the solvent in the Rayleigh‐Taylor instability, $\tau^{*}=12\mu \gamma /\rho^{2}g^{2}h_{0}^{3}$[31], which are longer than 100s in this work. The liquid film thickness was measured by deflectometry using a Nikon D5500 with AF‐P NIKKOR 18–55 mm lens. Images were recorded with 30 frames per second and analyzed using MATLAB every 15th frame (details in Note S2, Supporting Information).

### Statiscal Analysis

All data processing and statistical analysis were performed using MATLAB, and all experimental results were shown as mean ± SD. For Figure 3, thickness time series were extracted at n=13 spatial points and the fast Fourier transform (FFT) was computed for each point; the spectra were then summarized across the 13 points (see Note S3 for FFT details, Supporting Information). For Figures 4 and 5, each experiment yielded a single growth rate from the earliest observed instability event in a given film. The experiments were repeated n=7 times; the minimum and maximum values were excluded as outliers, and the remaining n=5 repeats were used for reporting.

## Conflicts of Interest

The authors declare no conflict of interest.

## Supporting information


**Supporting File 1**: advs73875‐sup‐0001‐SuppMat.pdf.

**Supporting File 2**: advs73875‐sup‐0002‐VideoS1.mp4.

**Supporting File 3**: advs73875‐sup‐0003‐VideoS2.mp4.

**Supporting File 4**: advs73875‐sup‐0004‐VideoS3.mp4.

**Supporting File 5**: advs73875‐supy0005‐VideoS4.mp4.

**Supporting File 6**: advs73875‐sup‐0006‐VideoS5.mp4.

## Data Availability

The data that support the findings of this study are available from the corresponding author upon reasonable request.
