# Supplementary material for: Evaporation‐Driven Solutal Marangoni Control of Rayleigh–Taylor Instability in Inverted Films
Source: Adv Sci (Weinh). 2026 Jan 29;13(20):e20343. doi: 10.1002/advs.202520343 (PMC13067858; doi:10.1002/advs.202520343)
Supplement: Supplementary file 1 — Supporting File 1: advs73875‐sup‐0001‐SuppMat.pdf. [file ADVS-13-e20343-s002.pdf]

Supporting Information

Evaporation-Driven Solutal Marangoni Control of Rayleigh–Taylor Instability in Inverted Films

*Minwoo Choi Hyejoon Jun Hyoungsoo Kim**

M. Choi, H. Jun, H. Kim
Department of Mechanical Engineering, KAIST, Daejeon, 34141, South Korea
Email Address: hshk@kaist.ac.kr

Contents

Figure S1. Theoretical growth rate from linear stability analysis.
Figure S2. Oscillatory mode of the instability determined from the numerical solution.
Figure S3. Measurement of evaporation rate via interferometry.
Figure S4. Comparison of the morphology of final dried thin films from water-based paint and water-ethanol paint.
Figure S5. Film thickness measurement by deflectometry.
Figure S6. Calibration of deflectometry.
Figure S7. Frequency measurement of oscillatory mode in a mixture of 80 wt% ethylene glycol and 20 wt% 2-butanol.
Figure S8. Evaporation rate measurement by Mach-Zehnder interferometry.
Supplementary Note S1. Detailed derivation of the analytical solutions.
Supplementary Note S2. Film thickness measurement
Supplementary Note S3. Frequency measurement
Supplementary Note S4. Estimation of evaporation speed
Supplementary Note S5. Numerical solutions
Supplementary Note S6. Thermophysical properties
Table S1. Dimensionless numbers of mixtures for the oscillatory mode.
Table S2. Dimensionless numbers of mixtures for the destabilized monotonic mode.
Table S3. Dimensionless numbers of mixtures for the stabilized monotonic mode.

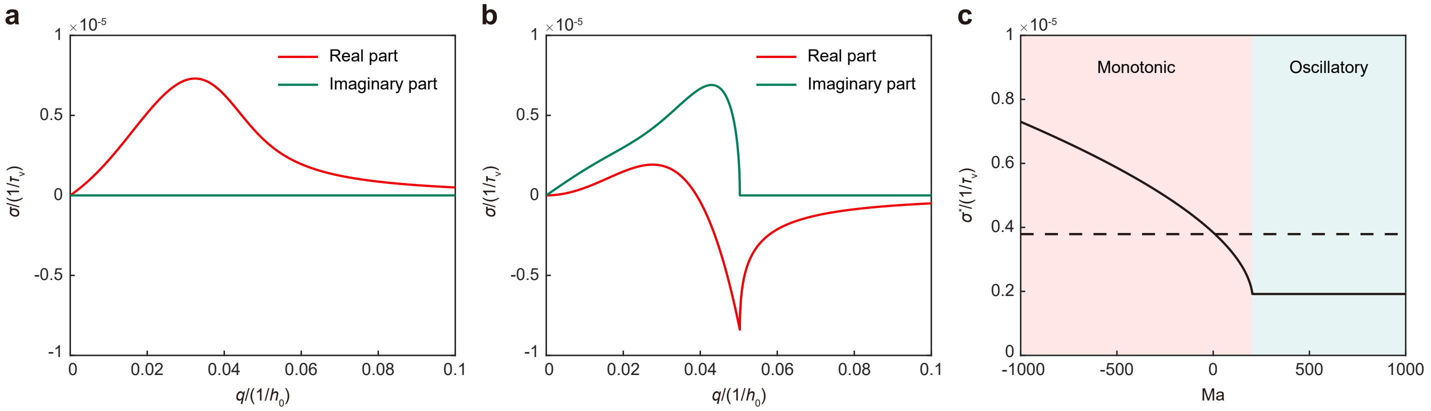

Figure S1: Theoretical growth rate from linear stability analysis. a-b) Real (red) and imaginary (green) parts of the dimensionless linear growth rate σ as a function of wave number q, for Marangoni numbers (a) Ma = −1000 and (b) Ma = 1000. c), Maximum linear growth rate σ^* versus the solutal Marangoni number Ma . The dashed line represents the growth rate without the Marangoni effect. The transition point ($\zeta = 0$) marks the onset of oscillatory instability. All results correspond to a binary mixture of ethylene glycol (80 wt%) and 2-butanol (20 wt%), with time and length scales nondimensionalized by the initial film thickness (h_0) and the viscous time scale ($\tau_v = h_0^2/\nu$), respectively.

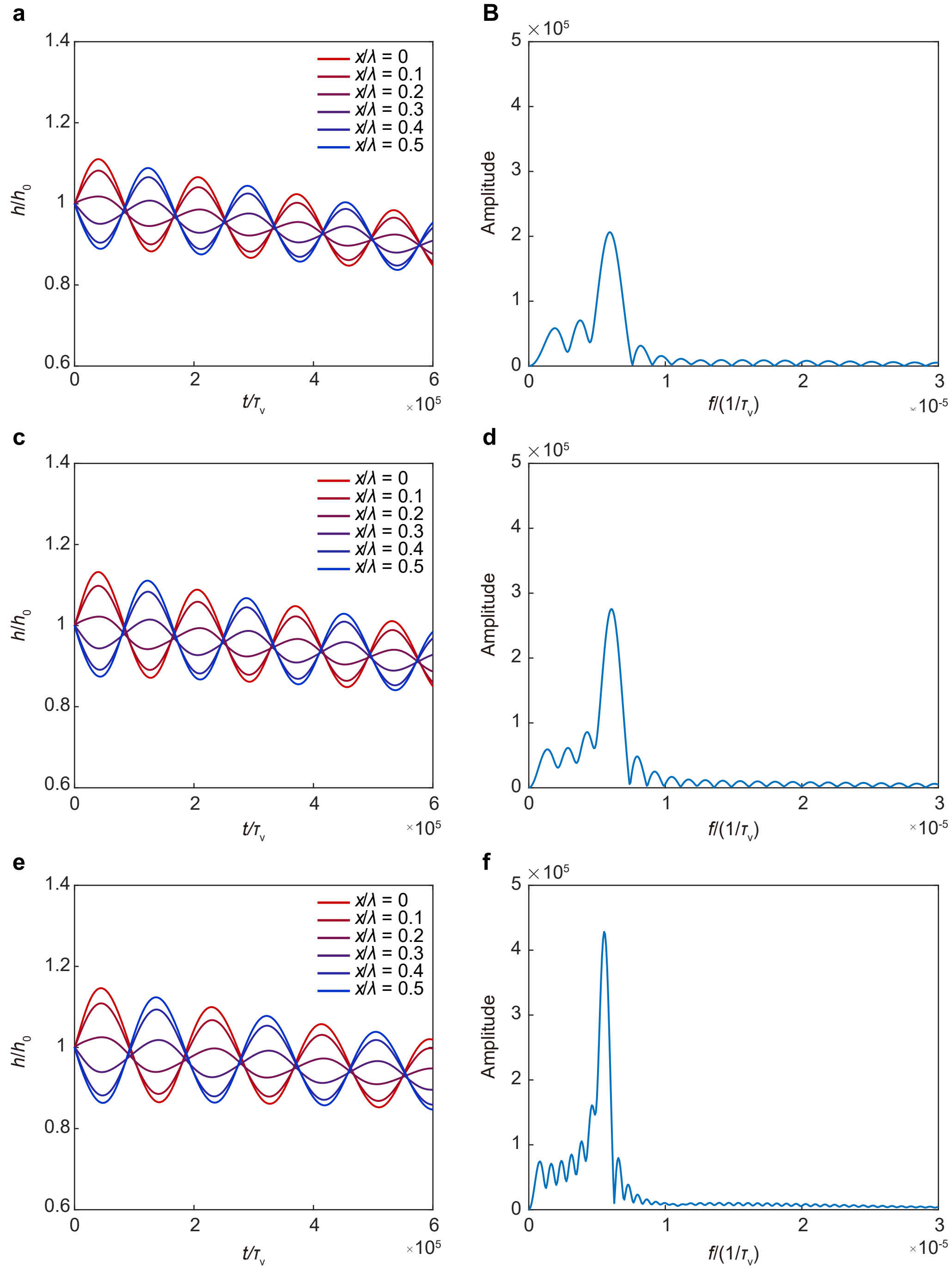

Figure S2: Oscillatory mode of the instability determined from the numerical solution. (Left) Time evolution of the dimensionless film thickness, h/h_0, at six spatial locations, x/λ = [0, 0.1, 0.2, 0.3, 0.4, and 0.5]. Both film thickness and time are nondimensionalized by the initial film thickness (h_0) and the viscous time scale ($\tau_v = h_0^2/\nu$), respectively. (Right) Fast Fourier transform (FFT) of the film thickness data shown in (left), highlighting a dominant oscillation frequency associated with the predicted interfacial instability. a-b): The mixture of 80 wt% ethylene glycol and 20 wt% 2-butanol. c-d): The mixture of 80 wt% ethylene glycol and 20 wt% isopropanol. e-f): The mixture of 80 wt% ethylene glycol and 20 wt% ethanol.

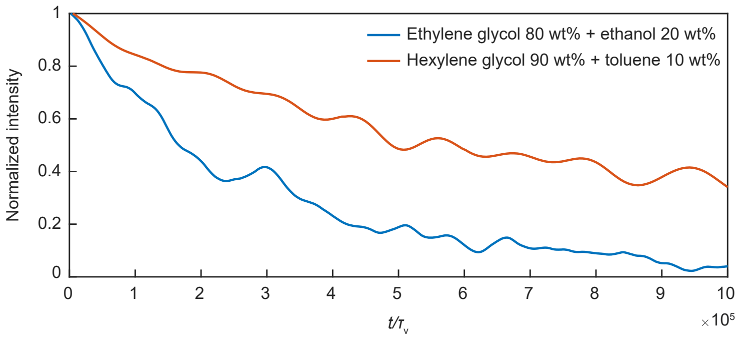

Figure S3: Measurement of evaporation rate via interferometry. Normalized intensity at the interface over dimensionless time, t/τ_ν, measured using interferometry for two binary mixtures: ethylene glycol (80 wt%) + ethanol (20 wt%) and hexylene glycol (90 wt%) + toluene (10 wt%). The intensity variation reflects the phase shift of the interference pattern induced by vapor generated during evaporation. The time is nondimensionalized by the viscous time scale, $\tau_\text{v} = h_0/\nu$. Further experimental details are provided in Supplementary Note 4 and Figure S8.

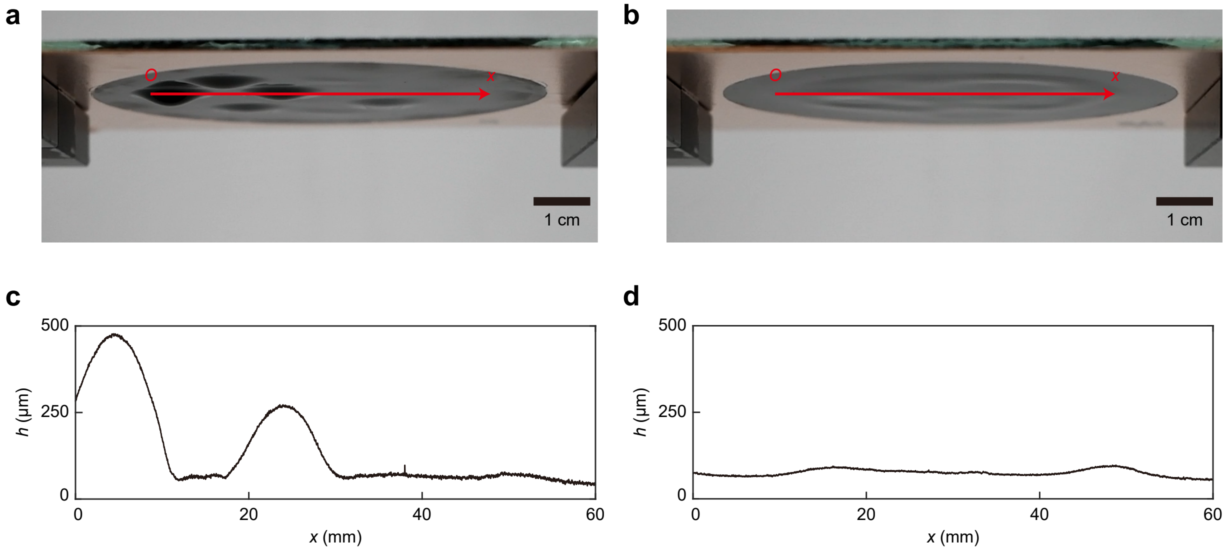

Figure S4: Comparison of the morphology of final dried thin films from water-based paint and water-ethanol paint. a-b) Suspended paint films after 30 minutes of evaporation. c-d) Final thickness profiles after the evaporation measured along the red lines indicated in (a) and (b), respectively. Paint compositions: water (40 wt%) with pigment (60 wt%) for (a) and (c), and water (36 wt%) with ethanol (4 wt%) and pigment (60 wt%) for (b) and (d). For more details, see Video S5. Thickness profiles measured with a confocal laser scanning microscope (VK-X1050, Keyence).

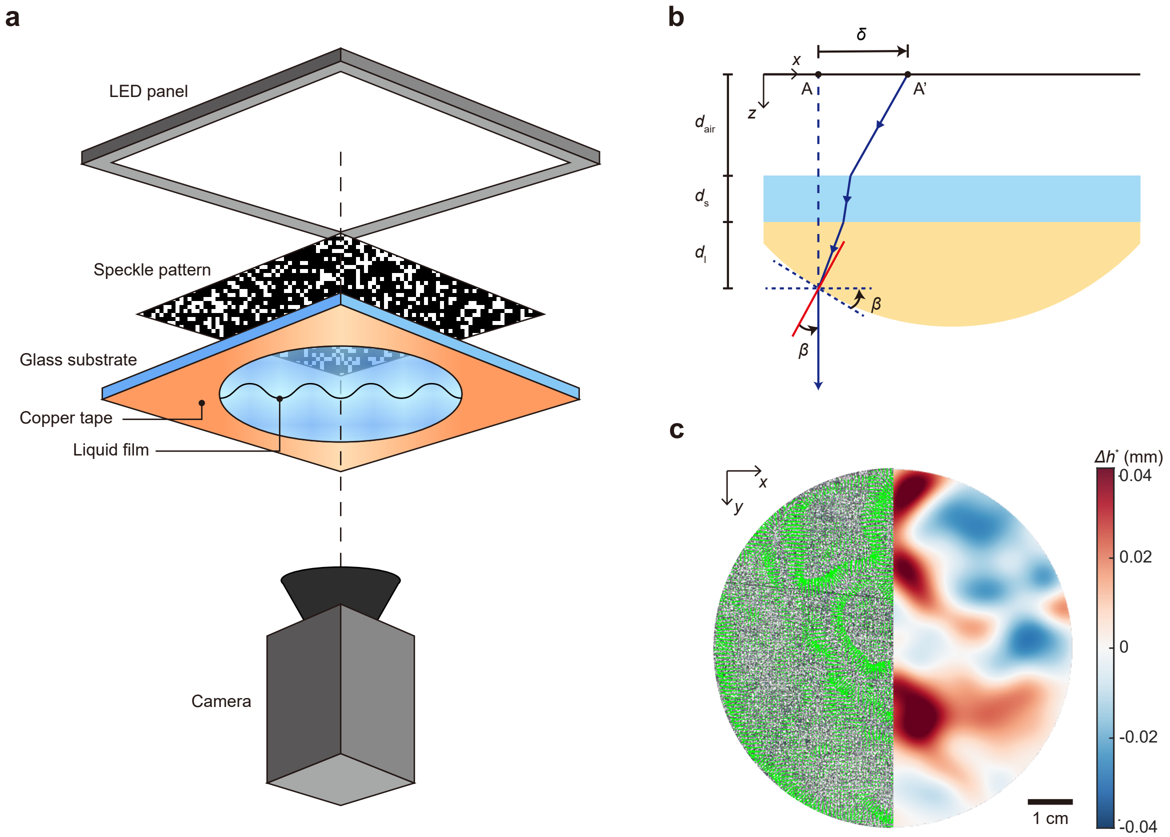

Figure S5: Film thickness measurement by deflectometry. a) Schematic of the experimental setup, deflectometry. The liquid film is surrounded by the copper tape. b) A light path refracted by the deformed interface. c) (Left) The vector of the displacement, δ deformed by the interface using the auto-correlation method. (Right) The contour of the film thickness reconstructed from the displacement vector.

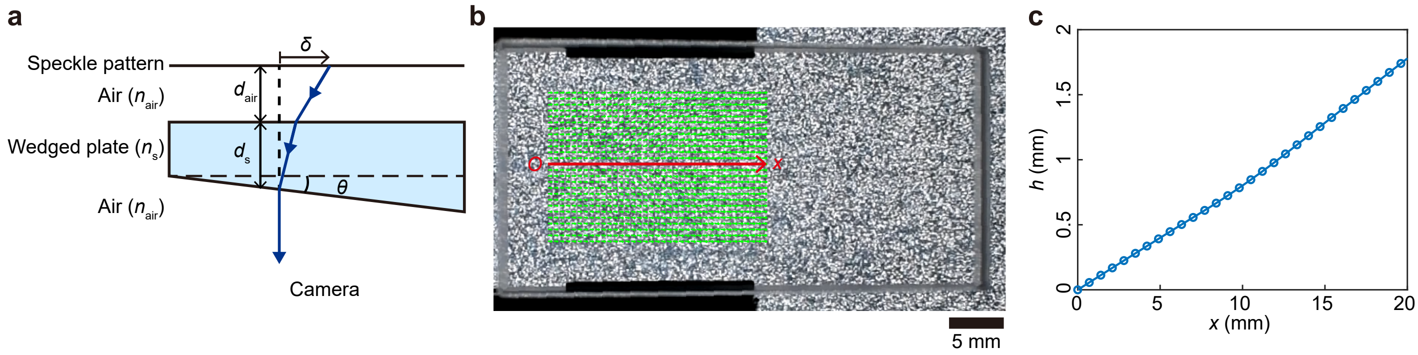

Figure S6: Calibration of deflectometry. a) Side-view schematic showing the refraction of light through a wedged plate with a wedge angle, $\theta = 5°$. b) Speckle pattern deformed by the wedged plate. The degree of deformation, indicated by green arrows, was measured using the auto-correlation method. c) The reconstructed interface profile along the red arrows in (b).

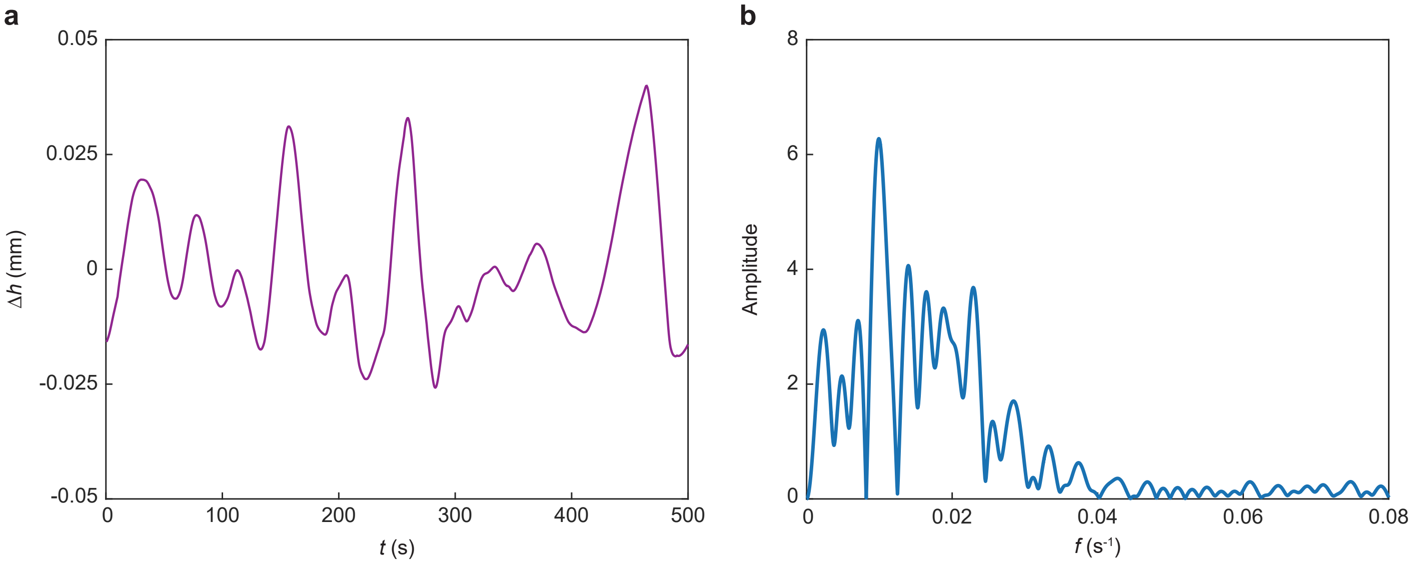

Figure S7: Frequency measurement of oscillatory mode in a mixture of 80 wt% ethylene glycol and 20 wt% 2-butanol. a) The film thickness over time at one point. b) Fast Fourier Transform (FFT) of the film thickness from (a).

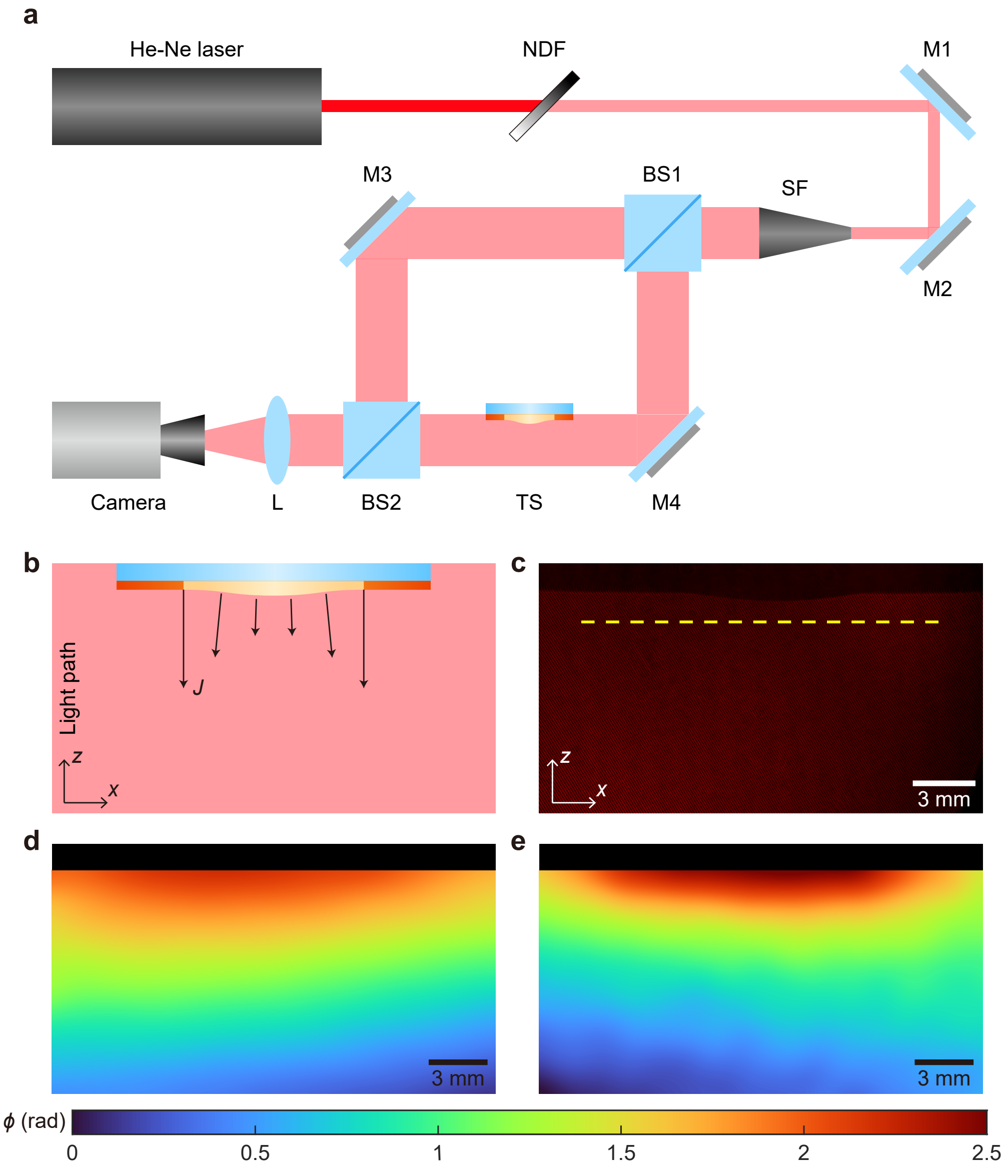

Figure S8: Evaporation rate measurement by Mach-Zehnder interferometry. a) Schematic of the Mach-Zehnder interferometer, where NDF is the neutral density filter, M is the mirror, SF is the spatial filter, TS is the test section and L is the lens. b) Schematic of the evaporating liquid film in the test section. c) Raw image of the evaporating liquid film with an interference pattern. The yellow dashed line (length = 25 mm) is 1.5 mm from the liquid film. d-e) Contour maps of phase-shift data of the mixtures: The mixture of ethylene glycol 80 wt% and 20 wt% ethanol for (d) and the mixture of 90 wt% hexylene glycol and 10 wt% toluene for (e).

Supplementary Note S1

Detailed derivation of the analytical solutions

Analytical solution of the oscillatory mode

To derive the coupled evolution equations for the thin film thickness and mass fraction in the film (Equations 1 and 2 in the main text), we used the Navier-Stokes, energy, and species transport equations, along with the boundary conditions. In particular, we focused on the dominant contributions from gravity, capillarity, and the solutal Marangoni effect to investigate Rayleigh–Taylor instability in binary mixtures.

For nondimensionalization, the initial film thickness and the horizontal length scale are used for length, $\tilde{x} = \frac{x}{\lambda}$, $\tilde{z} = \frac{z}{h_0}$, and $\tilde{h} = \frac{h}{h_0}$; the viscous scales are used for velocity, time, and pressure, $\tilde{u} = \frac{u}{U}$, $\tilde{w} = \frac{w}{\varepsilon U}$, $\tilde{t} = \frac{t}{\lambda/U}$, and $\tilde{p} = \frac{p}{\mu U/h_0}$, where $U = \frac{\nu}{\lambda}$ is the characteristic velocity; $\Delta T = T_s - T_g$ is used for temperature, $\tilde{T} = \frac{T-T_g}{\Delta T}$, where T_s and T_g are the temperatures of the substrate and the gas phase, respectively. Especially, the value of ΔT was determined by fitting the experimentally observed frequencies and growth rates, as it is too small to measure experimentally when the gas layer thickness significantly exceeds the liquid layer [1]. The evaporative mass flux is defined as $\tilde{J} = \frac{h_0 \mathcal{L}}{k\Delta T} J$. Also, the mass fraction is approximated using the vertical averaging (VA) approximation, $c(\tilde{x}, \tilde{z}, \tilde{t}) = C(\tilde{x}, \tilde{t}) + \varepsilon^2 \overline{\mathrm{Pe}} C_1(\tilde{x}, \tilde{z}, \tilde{t})$, where $\overline{\mathrm{Pe}} = \varepsilon^{-1}\mathrm{Pe}$.

Under the lubrication approximation and neglecting inertial terms, we simplified the governing equations for continuity, momentum, energy, and species transport of component B:

$$\tilde{u}_{\tilde{x}} + \tilde{w}_{\tilde{z}} = 0, \tag{S1}$$

$$-\tilde{p}_{\tilde{x}} + \tilde{u}_{\tilde{z}\tilde{z}} = 0, \tag{S2}$$

$$-\tilde{p}_{\tilde{z}} + \overline{\mathrm{Ga}} = 0, \tag{S3}$$

$$\tilde{T}_{\tilde{z}\tilde{z}} = 0, \tag{S4}$$

$$C_{\tilde{t}} + \tilde{u} C_{\tilde{x}} = \frac{1}{\overline{\mathrm{Pe}}} C_{\tilde{x}\tilde{x}} + C_{1,\tilde{z}\tilde{z}}, \tag{S5}$$

where $\overline{\mathrm{Ga}} = \varepsilon^{-1}\mathrm{Ga}$.

At the substrate ($\tilde{z} = 0$), no-slip, no-penetration, and constant temperature conditions were imposed:

$$\tilde{u} = \tilde{w} = 0, \quad \tilde{T} = 1. \tag{S6}$$

At the interface ($\tilde{z} = \tilde{h}$), the mass, energy, and species balances were derived:

$$\overline{\mathrm{E}}\tilde{J} = \tilde{w} - \tilde{h}_{\tilde{t}} - \tilde{u}\tilde{h}_{\tilde{x}}, \tag{S7}$$

$$\tilde{J} = -\tilde{T}_{\tilde{z}}, \tag{S8}$$

$$C_{1,\tilde{z}} = \overline{\mathrm{E}}(C - 1)\tilde{J}, \tag{S9}$$

where $\overline{\mathrm{E}} = \varepsilon^{-1}\mathrm{E}$. Furthermore, the normal and tangential stress balances at the interface were

$$\tilde{p} = -\frac{1}{\overline{\mathrm{Ca}}}\tilde{h}_{\tilde{x}\tilde{x}}, \tag{S10}$$

$$\tilde{u}_{\tilde{z}} = -\frac{\overline{\mathrm{Ma}}}{\mathrm{Pr}}C_{\tilde{x}}, \tag{S11}$$

where $\overline{\mathrm{Ca}} = \varepsilon^{-3}\mathrm{Ca}$, and $\overline{\mathrm{Ma}} = \varepsilon^{-1}\mathrm{Ma}$. Lastly, the evaporative mass flux was given by the nondimensionalized Hertz-Knudsen relation:

$$\mathrm{K}\tilde{J} = C\tilde{T}. \tag{S12}$$

The temperature profile and evaporative mass flux were derived by solving Eq. S4 and S8 with the constant wall temperature condition and the evaporative mass flux Equation S12:

$$\tilde{T} = 1 - \tilde{J}\tilde{z}, \tag{S13}$$

$$\tilde{J} = \frac{C}{\mathrm{K} + C\tilde{h}}. \tag{S14}$$

Using the continuity Eq. S1 and the momentum Eq. S2 and S3, along with the boundary conditions including the wall boundary condition and the stress balances (Equation S10 and S11), we derived the following expression for the velocity field:

$$\tilde{u} = \frac{\tilde{p}_{\tilde{x}}}{2}(\tilde{z}^2 - 2\tilde{h}\tilde{z}) - \frac{\overline{\mathrm{Ma}}}{\mathrm{Pr}}C_{\tilde{x}}\tilde{z}, \tag{S15}$$

$$\tilde{w} = -\frac{\tilde{p}_{\tilde{x}\tilde{x}}}{6}(\tilde{z}^3 - 3\tilde{h}\tilde{z}^2) + \frac{\tilde{p}_{\tilde{x}}\tilde{h}_{\tilde{x}}}{2}\tilde{z}^2 + \frac{\overline{\mathrm{Ma}}}{2\mathrm{Pr}}C_{\tilde{x}\tilde{x}}\tilde{z}^2, \tag{S16}$$

where $\tilde{p}_{\tilde{x}} = -\overline{\mathrm{Ga}}\tilde{h}_{\tilde{x}} - \frac{1}{\overline{\mathrm{Ca}}}\tilde{h}_{\tilde{x}\tilde{x}\tilde{x}}$. Finally, we have the evolution equations for the film thickness and mass fraction (Equations 1 and 2 in the main text) by integrating Equation S5 along the vertical direction (i.e., the z-direction) with the species balance Equation S9 and rescaling $\varepsilon \rightarrow 1$.

To assess the stability of the base state, the evolution equations were linearized by assuming perturbations of the form $\tilde{h} = 1 + \hat{H}e^{\tilde{\sigma}\tilde{t}+i\tilde{q}\tilde{x}}$ and $C = c_0 + \hat{C}e^{\tilde{\sigma}\tilde{t}+i\tilde{q}\tilde{x}}$. The evolution equations were linearized with respect to the film thickness and mass fraction, retaining the major terms - gravity, capillarity, and solutal Marangoni effect:

$$\tilde{\sigma}\hat{H} - \left(\frac{\mathrm{Ga}}{3}\tilde{q}^2 - \frac{1}{3\mathrm{Ca}}\tilde{q}^4\right)\hat{H} + \frac{\mathrm{Ma}}{2\mathrm{Pr}}\tilde{q}^2\hat{C} = 0, \tag{S17}$$

$$\tilde{\sigma}\hat{C} - \frac{\mathrm{E}(1-c_0)(\mathrm{K}/c_0+2)}{(\mathrm{K}/c_0+1)^2}\hat{H} + \frac{1}{\mathrm{Pe}}\tilde{q}^2\hat{C} = 0. \tag{S18}$$

By solving the coupled equations for $\hat{H}$ and $\hat{C}$, we derived a quadratic equation for the linear growth rate ($\tilde{\sigma}$), neglecting higher-order terms based on the magnitude of dimensionless parameters for the mixture (summarized in Supplementary Note S6 and Table S1-S3):

$$\tilde{\sigma}^2 + \left(-\frac{\mathrm{Ga}}{3}\tilde{q}^2 + \frac{1}{3\mathrm{Ca}}\tilde{q}^4\right)\tilde{\sigma} + \frac{\mathrm{E}(1-c_0)(\mathrm{K}/c_0+2)}{2(\mathrm{K}/c_0+1)^2}\frac{\mathrm{Ma}}{\mathrm{Pr}}\tilde{q}^2 = 0. \tag{S19}$$

The analytical solution (Equation 3 in the main text) was obtained from the quadratic equation for $\tilde{\sigma}$. We determined $\Delta T = 1.7 \times 10^{-4}$ °C for the analytical solution, and $\Delta T = 5.4 \times 10^{-3}$ °C for the numerical solution,

with discrepancies arising from nonlinear effects.

Analytical solution for slowly evaporative mixtures

For binary mixtures with the extremely low evaporation number, a modified perturbation form should be applied, $C = c_0 + \hat{C}e^{i\tilde{q}\tilde{x}}$. By substituting this form into the evolution equations, we newly derived the equation instead of Equation S18:

$$-\frac{\mathrm{E}(1-c_0)(\mathrm{K}/c_0+2)}{(\mathrm{K}/c_0+1)^2}H + \frac{1}{\mathrm{Pe}}\tilde{q}^2C = 0. \qquad \text{(S20)}$$

This leads to a linear equation for the linear growth rate, as Equation S20 is independent of $\tilde{\sigma}$. Consequently, the linear growth rate (Equation 4 in the main text) can be derived by solving the coupled equations for $\hat{H}$ and $\hat{C}$, Equations S17 and S20. The temperature differences used in the rescaling were $\Delta T = 1.2\times10^{-7}$ °C for the silicone oil-based mixtures and $\Delta T = 2.9\times10^{-8}$ °C for the hexylene glycol-based mixtures. This supports the assumption that thermal Marangoni effects can be neglected in the present system, as the extremely low temperature differences used in the rescaling render thermally induced surface tension gradients insignificant.

Supplementary Note S2

Film thickness measurement

Set-up and principle of deflectometry

We employed deflectometry to measure the film thickness, which detects image distortion in a background image (speckle pattern). The pattern was created by printing on an OHP film, with each pixel set to 0.15 mm. For the experimental setup of deflectometry, the liquid film was positioned between a speckle pattern and a camera for the bottom view imaging, as shown in **Figure S5**a. It is especially important to ensure that the pattern and the substrate remain parallel to the ground, using a level box for accuracy. The interface of the liquid film, deformed by gravity, acts as a variable-thickness lens, distorting the speckle pattern. The camera monitored this distortion with a frame rate of 30 fps while the Rayleigh-Taylor instability of the liquid film occurred. The light is refracted by the deformed interface, as shown in **Figure S5**b. The displacement of the pattern, δ follows Snell's law [2] as

$$\delta = \beta(n_{\text{air}} - n_l)(d_s/n_s + d_{\text{air}}/n_{\text{air}}) \tag{S21}$$

when $|\beta| \ll 1$, where β is the local slope of the interface, $n_{\text{air}} = 1$, $n_s = 1.52$ and n_l are the refractive indices of air, the glass substrate and solvents and $d_s = 2\,\text{mm}$ and $d_{\text{air}} = 5\,\text{mm}$ are the thickness of the glass substrate and air layer, respectively. The refractive indices of binary mixtures are determined from the mass-averaged mixing rule.

The displacement was measured by comparing a reference image with deformed images, which had been used for the auto-correlation method [3]. This is conducted over a range of 20 minutes which is sufficient to capture the full evolution of the instability. The image at the initial state (undeformed image) was used as the reference and was compared with images captured at sequential time points (deformed images). Finally, we got the displacement vector at each point that was obtained with a resolution of 8 pixels by comparing the reference and deformed images, as shown in **Figure S5**c. This setup enables measurements with a minimum slope of approximately 0.02° due to the limited resolution of the camera (1920 × 1080). This sensitivity can increase as the distance between the pattern and the liquid film with an expense of low accuracy.

Using Equation S21, the local slope at each point was calculated from the measured displacement. The film thickness was reconstructed by integrating the local slope, as shown in **Figure S5**c, assuming that the thickness of the liquid film in contact with the tape wall is equal to the thickness of the tape wall ($h_0 = 80\,\mu\text{m}$).

Calibration using wedged plate

For calibration, the wedged plate with a 5° wedge angle (uncoated UV fused silica, Thorlabs) was used as the substrate at the same position. To minimize errors caused by variations in substrate thickness, only a specific region (length along $x = 20\,\text{mm}$), as shown in **Figure S6**a, was extracted to measure the displacement of the deformed pattern. The substrate thickness within this region was assumed to remain constant at its average value of 7 mm. Using this assumption, the interface along the red line in **Figure S6**b was reconstructed, as illustrated in **Figure S6**c, resulting in a mean interfacial slope of 5.07°, which closely matches the actual slope value.

Supplementary Note S3

Frequency measurement

In the previous section, the contour of the film thickness was calculated at each time. Some of the binary mixtures exhibited oscillatory motions, which required frequency measurement to characterize the instability. To measure the frequency, the variation in the film thickness (Δh) at one point was plotted over time, as shown in Fig. S7A. Here, the film thickness was measured every 0.5 seconds for up to 500 seconds, which is sufficient to capture the oscillation frequency since it covers approximately five full oscillation periods. The dominant frequency was determined by applying Fast Fourier Transform (FFT) to the film thickness profile, as shown in **Figure S7**b, using 2^{27} samples. To determine the characteristic oscillation frequency of the entire film, the same procedure of film thickness measurement and FFT analysis was applied to 13 points in the inset of **Figure 3**d in the main text. The average of these FFT results was plotted, as shown in **Figure 3**d, and the most dominant value was determined as the oscillation frequency of the mixture.

Supplementary Note S4

Direct measurement of the evaporated vapor distribution by the laser interferometry

Binary mixtures show different modes of Rayleigh-Taylor instability depending on the evaporation number, $E = k\Delta T/\rho\nu\mathcal{L}$, which represents the ratio of the viscous time scale to the evaporative time scale. We verified that mixtures with lower E evaporate more slowly by measuring the vapor distribution around the film over time. We employed a Mach-Zehnder interferometer setup to visualize the vapor distribution, as depicted in **Figure S8**a. In this MZ interferometry, a He-Ne laser (JDL Uniphase) with a wavelength of 632.8 nm and an output power of 10 mW first passed through the neutral density filter (NDF) to adjust its brightness. The laser was expanded by a spatial filter (SF) and then split into two ways by the beam splitter (BS1), which were later recombined at BS2. One beam passed through the air without any flow, while the other passed through the test section (TS) containing the upside-down film evaporated as shown in **Figure S8**b. We adjusted the angle of the mirror (M3) to observe the interference pattern caused by the path difference between the two beams, as shown in **Figure S8**c. This regular interference pattern can be deformed by refractive index changes such as evaporated vapor from the film. The reference (undeformed) and deformed patterns were reconstructed into phase-shift data (ϕ) using a two-dimensional Fourier transform [4] as shown in **Figure S8**d and e. To measure the evaporation speed, the sum of the phase shift along the yellow dashed line (length = 25 mm) which has the same length as the film diameter in **Figure S3**c was plotted over time. Here, we placed the yellow dashed line (1.5 mm away from the interface) as close to the interface as possible since measurements closer to the interface better reflect the local evaporation speed without significant lateral diffusion. The sum on the yellow dashed line is normalized by its maximum value, and time nondimensionalized using the viscous time scale ($\tau_\nu = h_0^2/\nu$). However, in cases where the geometry loses its axisymmetry, the interferometer can only provide a two-dimensional projection of the phase shift, and not the local evaporative flux. Instead, we assumed a one-sided evaporation model based on the Biot number condition in our experiments [5].

Supplementary Note S5

Numerical solutions

In the main text, evolution equations for the oscillatory mode were obtained (Equations 1 and 2 in the main text). The one-dimensional equations for the oscillatory mode were solved using the finite difference method. Calculations were performed for a single wavelength with periodic boundary conditions in the x-axis. The wavenumber ($\tilde{q}$) was set to the most unstable value defined as $(\mathrm{GaCa}/2)^{0.5}$, which is determined from the analytical solution of the linear growth rate. The computational domain along the x-axis was discretized into 100 points, and the evolution of the solution in time was computed with a time step of 0.1 using the Euler method. The initial conditions for the equations were specified as:

$$\tilde{h}(\tilde{x}, 0) = H_0 - \hat{H}\cos(\tilde{q}\tilde{x}), \tag{S22}$$

$$C(\tilde{x}, 0) = C_0 - \hat{C}\cos(\tilde{q}\tilde{x}), \tag{S23}$$

where $H_0 = 1$ represents the normalized initial thickness of the film and $C_0 = 0.2$ denotes the initial concentration of the binary mixture. To illustrate experiments of the oscillatory mode, we used the initial conditions:

$$\tilde{h}(\tilde{x}, 0) = 1 - 10^{-3}\cos(\tilde{q}\tilde{x}), \tag{S24}$$

$$C(\tilde{x}, 0) = 0.2 - 10^{-3}\cos(\tilde{q}\tilde{x}). \tag{S25}$$

The results of the numerical calculation are shown in **Figure S2**. The frequency was determined by the equation in the main text, but the mixture with the low evaporation number always shows a high amplitude of the oscillation and a low mean slope. Their oscillations were analyzed by the FFT (Fast Fourier Transform), which is the same method as described in Supplementary Note S3.

Supplementary Note S6

Section 6. Thermophysical Properties

All thermophysical properties used in Table S1-S3 are referred by the handbook [6].

Table S1: Dimensionless numbers of mixtures for the oscillatory mode.

Solvent (80 wt%)	Solute (20 wt%)	E ($\times10^{-9}$)	K ($\times10^{-2}$)	Ma ($\times10^{3}$)	Pe ($\times10^{5}$)	Ga ($\times10^{-2}$)	Ca ($\times10^{-2}$)	Pr ($\times10^{2}$)
Ethylene glycol	Dioxane	7.41	1.74	1.06	2.24	3.49	4.38	1.37
	2-butanol	4.96	1.79	1.67	2.54	3.03	5.04	1.54
	Isopropanol	4.22	0.69	2.00	2.44	3.11	4.96	1.56
	Ethanol	3.41	0.48	1.81	1.93	3.20	4.79	1.43
	Methanol	2.76	0.25	1.68	1.39	3.26	4.68	1.38

Table S2: Dimensionless numbers of mixtures for the destabilized monotonic mode.

Solvent (90 wt%)	Solute (10 wt%)	E ($\times10^{-12}$)	K ($\times10^{-1}$)	Ma ($\times10^{2}$)	Pe ($\times10^{5}$)	Ga ($\times10^{-2}$)	Ca ($\times10^{-1}$)	Pr ($\times10^{2}$)
Silicone oil 20cst	Dioxane	2.58	1.06	-6.04	3.37	1.55	1.78	1.93
	O-xylene	2.40	3.16	-4.52	4.33	1.51	1.82	1.97
	Toluene	2.72	1.50	-3.56	3.58	1.51	1.83	1.95
	Butyl acetate	2.65	2.13	-2.11	3.57	1.51	1.86	1.98
	Ethyl acetate	2.73	0.48	-1.33	3.08	1.52	1.86	1.96

Table S3: Dimensionless numbers of mixtures for the stabilized monotonic mode.

Solvent (90 wt%)	Solute (10 wt%)	E ($\times10^{-13}$)	K ($\times10^{-1}$)	Ma ($\times10^{2}$)	Pe ($\times10^{6}$)	Ga ($\times10^{-3}$)	Ca ($\times10^{-1}$)	Pr ($\times10^{2}$)
Hexylene glycol	Dioxane	3.70	1.13	0.16	1.16	4.61	3.83	5.70
	O-xylene	3.44	3.36	1.58	1.42	4.48	3.94	5.81
	Toluene	3.90	1.59	2.42	1.18	4.47	3.92	5.78
	Butyl acetate	3.80	2.26	3.77	1.18	4.48	3.98	5.82
	Ethyl acetate	3.92	0.51	4.46	1.01	4.50	3.98	5.78

References

[1] H. Machrafi, A. Rednikov, P. Colinet, P. C. Dauby, *J. Colloid Interface Sci.* **2010**, *349*, 1 331.

[2] J. M. Burgess, A. Juel, W. McCormick, J. Swift, H. L. Swinney, *Physical Review Letters* **2001**, *86*, 7 1203.

[3] R. J. Adrian, J. Westerweel, *Particle Image Velocimetry*, 30. Cambridge university press, **2011**.

[4] M. Kuk, J. Pyeon, H. Kim, *Journal of Colloid and Interface Science* **2023**, *652* 646.

[5] H. Machrafi, A. Rednikov, P. Colinet, P. C. Dauby, *Phys. Rev. E* **2015**, *91* 053018.

[6] W. M. Haynes, *CRC Handbook of Chemistry and Physics*, CRC press, **2016**.
